# Intermittent preventive treatment with sulfadoxine-pyrimethamine does not modify plasma cytokines and chemokines or intracellular cytokine responses to *Plasmodium falciparum *in Mozambican Children

**DOI:** 10.1186/1471-2172-13-5

**Published:** 2012-01-26

**Authors:** Diana Quelhas, Laura Puyol, Llorenç Quintó, Tacilta Nhampossa, Elisa Serra-Casas, Eusébio Macete, Pedro Aide, Sergi Sanz, John J Aponte, Denise L Doolan, Pedro L Alonso, Clara Menéndez, Carlota Dobaño

**Affiliations:** 1 Centro de Investigação em Saúde da Manhiça (CISM), Manhiça, Mozambique; 2 Barcelona Centre for International Health Research (CRESIB), Hospital Clínic, Universitat de Barcelona, Spain; 3 Instituto Nacional de Saúde, Ministério da Saúde, Maputo, Mozambique; 4 Direcção Nacional de Saúde, Ministério da Saúde, Maputo, Mozambique; 5 Queensland Institute of Medical Research (QIMR), Brisbane, Australia

**Keywords:** cytokines, chemokines, IPTi, falciparum malaria, sulfadoxine-pyrimethamine

## Abstract

**Background:**

Cytokines and chemokines are key mediators of anti-malarial immunity. We evaluated whether Intermittent Preventive Treatment in infants with Sulfadoxine-Pyrimethamine (IPTi-SP) had an effect on the acquisition of these cellular immune responses in Mozambican children. Multiple cytokines and chemokines were quantified in plasma by luminex, and antigen-specific cytokine production in whole blood was determined by intracellular cytokine staining and flow cytometry, at ages 5, 9, 12 and 24 months.

**Results:**

IPTi-SP did not significantly affect the proportion of CD3+ cells producing IFN-γ, IL-4 or IL-10. Overall, plasma cytokine or chemokine concentrations did not differ between treatment groups. Th1 and pro-inflammatory responses were higher than Th2 and anti-inflammatory responses, respectively, and IFN-γ:IL-4 ratios were higher for placebo than for SP recipients. Levels of cytokines and chemokines varied according to age, declining from 5 to 9 months. Plasma concentrations of IL-10, IL-12 and IL-13 were associated with current infection or prior malaria episodes. Higher frequencies of IFN-γ and IL-10 producing CD3+ cells and elevated IL-10, IFN-γ, MCP-1 and IL-13 in plasma were individually associated with increased malaria incidence, at different time points. When all markers were analyzed together, only higher IL-17 at 12 months was associated with lower incidence of malaria up to 24 months.

**Conclusions:**

Our work has confirmed that IPTi-SP does not negatively affect the development of cellular immune response during early childhood. This study has also provided new insights as to how these cytokine responses are acquired upon age and exposure to *P. falciparum*, as well as their associations with malaria susceptibility.

**Trial Registration:**

ClinicalTrials.gov: NCT00209795

## Background

In 2009 there were an estimated 68,925,435 cases of malaria in the African region (78% of worldwide estimates) and 111,885 malaria deaths (91% of worldwide estimates) [1]. These figures demonstrate that *Plasmodium falciparum *malaria remains a major threat to the health of Africans, in particular children under 5 years of age. In malaria endemic areas, older children and adults develop immunity to severe morbidity and death, though remaining susceptible to infection [2,3]. Immunoglobulin passive transfer studies in humans suggest that antibodies are key mediators of naturally acquired immunity [4]. More recent data [5] suggest that cellular immunity also plays an important role in the protection against *P. falciparum *disease in humans [6].

Cytokines and chemokines are considered key mediators of anti-malarial immunity. For example, the production of cytokines and chemokines by activated leukocytes, including CD8+ and CD4+ T cells, mediate mechanisms such as phagocytosis and killing of parasitized erythrocytes [7] and inhibition of parasite growth and development inside hepatocytes [8].

It has been also shown that cytokines play an important role in the immunopathology of malaria and many field studies have described an association of specific cytokines with severity of disease, in particular IL-2 [9], IL-12 [10,11], IFN-γ [9,12], IL-1β [13], IL-6 [14,15], TNF [9,13-22], IL-4 [23-25], IL-10 [9,12,20,26,27], MIP-1β [28], and TGF-β [29].

There is less field data available on the relevance of individual cytokines in naturally acquired immunity to malaria, e.g. IL-12 [30-32], IFN-γ [30,32-36], TNF [30,34,37], or IL-10 [38]. Previous studies have investigated individual or a few cytokines but it is more likely that a network of cytokine and chemokines determines protection or susceptibility from *P. falciparum *infections [39]. Most studies of cytokine responses have been cross-sectionals done after the onset of symptoms and at the initial stages of clinical illness [40-43]. Only a few studies have assessed longitudinally the evolution of cellular immune responses to infection, and those have been mostly in adult populations [30,44,45]. Furthermore, while a number of studies have evaluated newborn cytokine responses in cord blood [46-54], very few have measured prospectively cellular immune responses to *P. falciparum *in infants [55] or children [30,44,56].

In the context of a randomized, placebo-controlled trial of intermittent preventive treatment in infants with sulfadoxine-pyrimethamine (IPTi-SP) in Mozambique [57], we have previously shown that this intervention had no impact on the antibody responses to *P. falciparum *erythrocytic stage antigens and to variant surface antigens in Mozambican infants, nor on the capacity of antibodies to inhibit parasite growth [58,59]. Only a handful of studies have evaluated the impact of any malaria control interventions on the development of cellular immune responses. In one study, continuous chemoprophylaxis for 3 years in Gambian children resulted in higher lymphoproliferative responses and IFN-γ production [60], and there was no clinical rebound of malaria one year after termination of prophylaxis (despite a decrease in anti-malarial antibody levels). In another study, permethrin-treated bednets showed a significant impact on percent lean body mass in Kenyan school children, thought to be due to decreased production of pro-inflammatory cytokines TNF, IL-1 and IL-6 in this group [61]. However, no study has yet assessed the impact on the child's cellular immunity of IPTi-SP, a safe and efficacious malaria control strategy consisting in the administration of a full dose of this antimalarial drug within the Expanded Program on Immunization (EPI) [62].

In the current study, we have determined prospectively the effect of IPTi-SP on levels of *P. falciparum *antigen-specific intracellular cytokines using flow cytometry in children up to 2 years of age. To partially overcome the difficulty to study this age group because of limited blood volumes, we used whole blood assays (WBA) instead of isolated peripheral blood mononuclear cells for measuring antigen-specific responses by intracellular cytokine staining (ICS). This approach also provides an environment more similar to that existing *in vivo*, and it is faster and more economical [53,63-65]. In addition, we have used luminex-based microsphere suspension array methods to measure multiple cytokines and chemokines free in small volumes of plasma [54,66]. Finally, we assessed the factors influencing the levels of cytokines and chemokines at different time points and the associations between these and the incidence of malaria in the first and second years of life. This type of work has not been as comprehensively conducted in this age group with a longitudinal design in previous field studies.

## Methods

### Study area

The study was conducted at the Centro de Investigação em Saúde da Manhiça, Manhiça District, southern Mozambique. This area is characterized by a perennial malaria transmission mostly due to *Plasmodium falciparum. Anopheles funestus *is the main vector, and the estimated average number of infective mosquito bites per person per year was 38 (IDRC and INDEPTH network 2002). For children in this area, SP showed a combined (early and late) therapeutic efficacy rate of 83%, with an *in vivo *parasitological sensitivity of 78.6% at day 14 [67].

### Study design

The efficacy study was an individually randomized, placebo-controlled trial [57]. Infants were recruited from those attending the EPI clinic to receive dose 2 of DTP/OPV/Hep B between September 2002 and February 2004. Treatment with SP or placebo was administered at 3, 4, 9 months of age alongside the routine vaccinations. Cross-sectional visits were scheduled at 5, 9, 12 and 24 months of age. For the ancillary immunological studies within IPTi, we included the last 501 children recruited in the main trial. At each cross-sectional visit, capillary blood collected by fingerprick was placed into EDTA microtainers (1 ml), to obtain plasma for extracellular cytokines, and into heparin microtainers (0.5 ml) for intracellular cytokine determinations. All immunological assays were performed by personnel who were blinded to the children's study group. Clinical surveillance for malaria morbidity was done through passive case detection. Written informed consent was obtained from all parents or guardians and ethical approval for the protocol was obtained from the ethics review committees of Mozambique and the Hospital Clinic, Barcelona, Spain. The trial registration number is NCT00209795 http://clinicaltrials.gov.

For intracellular cytokine staining (ICS) assays, among those recruited, children with the following criteria were selected: (i) having received all 3 doses of SP or placebo, (ii) having a full set of months 5, 9 and 12 blood samples or the least visits missing, and (iii) having an equal representation of SP- and placebo-recipients. Among those, 258 samples were analyzed from month 5, 208 samples from month 9 and 144 samples from month 12. Once month 24 samples were collected, 143 that had the previous set were analyzed.

For luminex assays, those children who came for all 4 visits or the least visits missing were prioritized, and plasmas were selected for analysis when there were sufficient volumes available: 229 children samples from month 5, 221 from month 9, 225 from month 12, and 158 from month 24.

### Multiplex flow cytometric assay to measure cytokines and chemokines in plasma

Plasma cytokines and chemokines IL-2, IL-12 (p70), IFN-γ, IL-1β, IL-6, TNF, IL-4, IL-5, IL-8, IL-13, IL-10, IL-7, IL-17, G-CSF, GM-CSF, MCP-1 (MCAF), MIP-1β were determined using a Bio-plex 100 System (Bio-Rad, CA, USA) and following manufacturer's instructions. According to supplier availability, two types of cytokine detection kits were used (10-plex and 17-plex). Most samples (all except those from 26 children) were tested using the 17-plex kit; the first 27 children (visits 5, 9, 12 months) were tested using the 10-plex kit (which did not contain IL-1β, IL-5, IL-7, IL-8, IL-12, IL-13 IL-17, G-CSF, GM-CSF).

Assays were conducted in 96-well Millipore multiscreen filter plates (Millipore, MA, USA), running in duplicate 8 standards and 40 plasmas per plate. Plasma samples previously stored at -80°C were thawed in a water bath at 37°C, and centrifuged at 1,000 g at 4°C for 10 min. Antigen-coated luminex beads were incubated with patient plasma, in conjunction with manufacturer's reagents. Plasma samples were diluted in Bio-Plex human sample diluent as 1:3 volumes (30 μl sample + 90 μl diluent). To each sample well, the following were added in a step wise manner: 50 μl premixed beads (1×), 50 μl of diluted standard or sample, and 25 μl Bio-Plex detection antibody (1×), and 50 μl streptavidin-PE preparation (1×). The mixture was incubated for 30 min in the dark, at room temperature, on a rotating platform. The wells were then washed 3 times to remove unbound excess serum using a vacuum filtration system and a final incubation was done with streptavidin-PE for 10 min at room temperature while shaking at 300 rpm. Beads were finally resuspended in 125 μl assay buffer, and shaken on a microplate shaker at 1,100 rpm at room temperature for 30 sec immediately before reading the plate on the Luminex 100, in accordance with manufacturer's instructions. Calibration was done using the CAL2 high RP1 target value. Concentrations of unknown cytokines were calculated by plotting unknowns against a 5-parameter logistic regression standard curve of 8 points that determine the limits of detection for each cytokine, expressed as pg/ml. The coefficient of variation (%CV, reproducibility between duplicate values) needed to be < 20% to accept the value for analysis. Results were analyzed using the Bio-Rad Bioplex Manager Software v4.0.

### Intracellular cytokine staining to measure *P. falciparum*-specific responses

**Figure 1:**
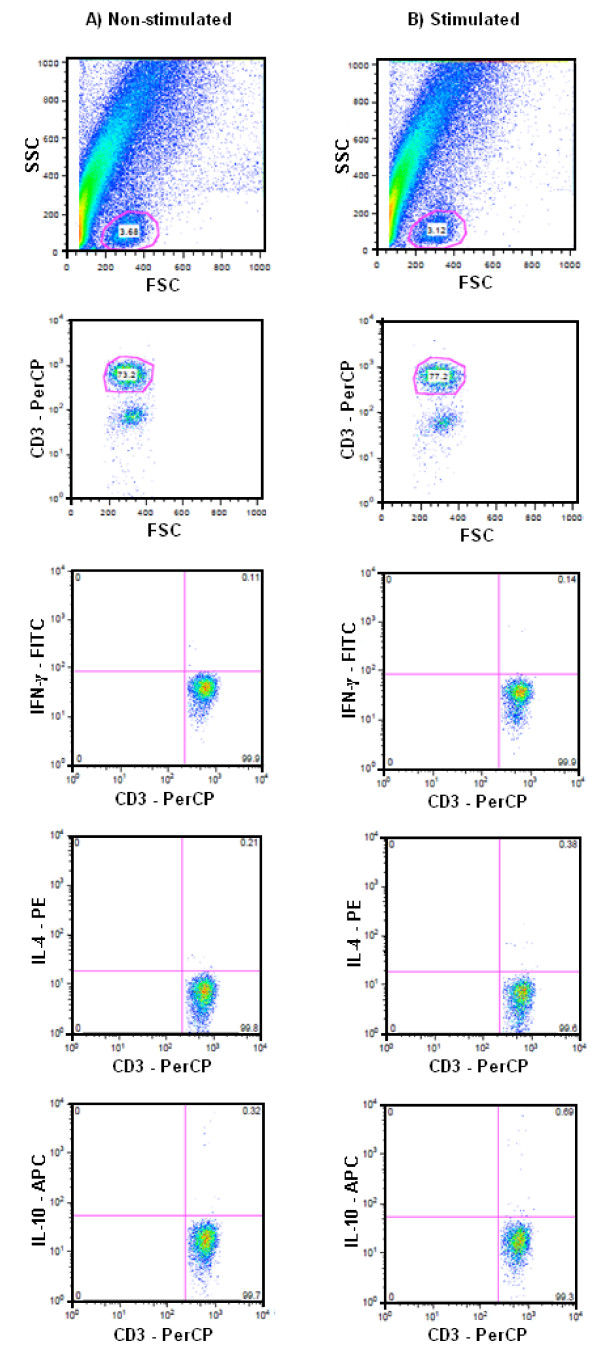
**Representative FACS plots showing flow cytometric detection of *P. falciparum *antigen-specific IFN-γ (FITC-labeled), IL-4 (PE-labeled) and IL-10 (APC-labeled) following intracellular cytokine staining of CD3^+ ^lymphocytes**. Non-stimulated control samples (A) were used to establish the threshold quadrants of background responses that were applied to quantify % of positive cytokine-producing cells in paired antigen-stimulated samples (B) of the same child. The UR quadrant represents CD3^+ ^cytokine^+ ^lymphocytes.

CD3+ specific intracellular cytokines IFN-γ, IL-4 and IL-10 were measured by ICS. Heparinized whole blood was aliquoted into two tubes to test in parallel the stimulated and the non-stimulated (negative/background control) samples. These were respectively incubated with 20 μl extract of *P. falciparum *schizont lysate at 1 × 10^8^schizonts/ml or 20 μl uninfected erythrocyte extract, both in the presence of 1 μg/ml co-stimulatory antibodies to CD28 (clone CD28.2) and CD49d (clone 9F10) (BD Biosciences, San Jose, CA) for 16 h at 37°C and 5% CO_2_. To block cytokine secretion, 1 μM Brefeldin A (GolgiPlug™, Pharmingen, San Diego, CA) was added during the final 4 h of incubation. To arrest activation and avoid loss of activated cells by adhesion to polystyrene tubes, 2 mM EDTA/PBS solution was added for 15 min at room temperature, and tubes were vigorously vortexed before and after the incubation. In order to lyse erythocytes and fix lymphocytes, 5 ml 1× FACS Lysing solution were added. These were vortexed, incubated for 10 min at room temperature, and immediately placed at -80°C. Frozen samples were thawed in batches at a later time for parallel processing and staining, hereby avoiding loss of function or increased background staining. Thawing was done by briefly placing samples in a 37°C water bath and immediately washing off lysing solution using 7 ml Cell Wash buffer. Samples with clumping/debris of dead cells were previously filtered through 70 μm cell sieve filters (Cell Strainer BD Falcon), and pellet was resuspended with a pipette in 2 ml permeabilizing solution before vortexing for 10 min at RT. Prior to staining, frozen cells were washed with 8 ml wash buffer and centrifuged at 1,500 rpm for 10 min at room temperature. Supernatant was decanted, residual pellet was resuspended in 500 μl wash buffer, and 100 μl of each sample were transferred to 96 well round bottom microtiter plates. Plates were centrifuged at 2,000 rpm for 5 min, then flicked to discard the supernatants and finally pellets were resuspended by vortexing before adding the staining antibodies. Samples were stained with a pool of fluorescence-conjugated antibodies: 5 μl CD3 PerCP (clone SK7), 7 μl IFN-γ FITC (clone 25723.11), 3 μl IL-4 PE (clone 8D4-8), and 4 μl IL-10 APC (clone JES3-19F1) (BD Biosciences, San Jose, CA). After incubating at room temperature in the dark for 30 min, samples were washed twice and supernatants discarded. Samples were brought up to a total volume of 300 μl by adding Cell Wash buffer before acquisition. Stained samples were acquired on a 4-color FACS Calibur (BD Biosciences) and 50,000-100,000 total events were collected. Isotype controls were included to evaluate autofluorescence and to assist in setting the quadrants. The data were analyzed with CELLQuest software (BD Biosciences). Lymphocytes were gated using FSC vs. SSC plots and then SSC vs. CD3+ T cells plots were used to isolate CD3+ cells (Figure 1). We analyzed samples in which > 1,000 CD3+ events could be acquired, and in which defined lymphocyte and CD3+ cell populations could be identified, thus excluding those with compromised cell recovery that could confound the readings. Each intracellular cytokine was assessed within its respective fluorescence channel (PerCP vs. FITC or PE or APC). Non-stimulated control samples were used to establish the threshold quadrants of background responses that were applied to quantify % of positive cytokine-producing cells in paired antigen-stimulated samples; results were expressed as the percentage of cytokine (IL-4, IL-10, IFN-γ)-producing CD3+ cells. Samples were considered positive if there was a true difference in response proportions (95% confidence interval [CI]) between the response to test antigens (stimulated) and the background responses to the non-stimulated samples: if the CI was entirely below 0.05%, the response was negative; if the CI was entirely above 0.05%, the response was positive; if the CI overlapped with 0.05%, the response was indeterminate.

### Definitions and statistical methods

Malaria infection was defined as the presence of asexual *P. falciparum *parasites of any density in a blood smear measured by microscopy and/or by PCR [59]. A clinical malaria episode was defined as a positive blood smear plus an axillary temperature of 37.5°C or history of fever within the prior 24 h. The sensitivity and specificity of these definitions are 100% and 84%, respectively, in infants and 100% and 79.4% in children 1-4 years old [68]. To estimate the incidences, the time at risk was calculated as the number of person years at risk (PYAR; episodes per 365.25 days) since the beginning of the time at risk until the end of follow-up, migration, death or withdrawn consent, whichever occurred first. Children did not contribute to the time at risk or the clinical malaria cases during the lag periods (an arbitrary lag of 21 days was applied after a case of clinical malaria) to account for the effective prophylactic time after antimalarial drug treatment. Months were defined as 30.4 days.

A Shapiro-Wilk normality test revealed a non-normal distribution of data, therefore all analyses were carried out in log scale (Ln) to normalise the data, and averages were expressed as Geometric Means (GM). A Pearson correlation coefficient was used to measure the degree of correlation among cytokines. Correlations were considered "low" if *Rho *< 0.33, "moderate" if *Rho *was between 0.33 and 0.66, or "high" if *Rho *> 0.66. Ratios between cytokines were obtained by dividing the values from each child, transforming that value to its natural log, then calculating the mean for the total number of children, and finally transforming that value to its exponential (GM). T-test and chi-square or fisher exact test were used to compare mean levels or prevalence (tertiles of distribution) respectively, between IPTi treatment groups. Unadjusted and adjusted linear regression models were also used to estimate the effect of IPTi-SP on the cytokine levels at each visit.

To identify what variables were independently associated with cytokine responses (intervention group [SP or placebo], age at visit, neighborhood of residence, current infection and the occurrence of previous clinical episodes [yes/no]), linear regression models with random intercept were estimated, assuming normal distribution of residuals. Selection of the variables for the final model was done through a stepwise procedure, where the criterion for including or excluding a variable was having a p-value ≤ 0.05 or ≥ 0.10 respectively from the Likelihood ratio test.

To evaluate the relationship between each of the individual cytokines at ages 5 and 12 months and the incidence of clinical malaria episodes from 5 to 12 or from 12 to 24 months, respectively, unadjusted and adjusted negative binomial regression models were estimated using a stepwise procedure. In the study of multiple episodes, the probabilities that the criteria to apply Poisson regressions are met are very low, and the phenomenon of over dispersion can occur, resulting in underestimation of CI. The negative binomial regression can estimate part of the variance that Poisson regression cannot identify, and it is exactly the same when there is no over dispersion. Cytokine responses were treated as continuous values, or categorized by tertiles (cut-offs in Additional file 1, Table S1). The incidence rate ratio (IRR) of children with cytokine levels in the highest tertile against those in the lowest tertile was estimated, as well as the IRR per 2-fold increase in the value of cytokines. Co-variates included were: sex, neighborhood of residence, treatment group and previous malaria episodes.

Two additional separate negative binomial regression models were estimated including (i) all the cytokine responses together and (ii) all the cytokine together plus antibody variables assessed in our previous studies (IgG against whole parasite and variant antigens on the surface of infected erythrocytes, IgG [total or isotypes] and IgM against the merozoite antigens AMA-1, EBA-175 and MSP-1_19_) [58,59] to identify what immunological variables were independently associated with the subsequent incidence of clinical malaria.

Data analysis was performed using Stata 11 for Windows (Stata Corporation, College Station, TX). Statistical significance was defined as a *P *of < 0.05. Crude *P *values reported in the manuscript were interpreted for internal coherence and consistency of results and biological plausibility. In addition, *P *values were adjusted statistically for multiple comparisons by the Bonferroni correction (*P *values not reported). Most of the associations were no longer statistically significant (*P *≥ 0.05) once adjusted for multiple comparisons, except for a few associations which remained significant (indicated in the text and tables).

## Results

The clinical and parasitological characteristics of the children included in this study are reported in Additional file 2, Table S2.

### Intracellular and plasma cytokine responses in relation to IPTi-SP

The administration of IPTi-SP did not significantly affect the proportion of CD3+ cells producing intracellular IFN-γ, IL-4 or IL-10 in peripheral whole blood samples stimulated ex-vivo with *P. falciparum *extract antigens, at any of the time points, neither by unadjusted analysis nor by adjusting for previous episodes of clinical malaria (Table 1).

**Table 1:** Comparison of levels of intracellular (*P. falciparum*-specific) and plasma cytokines and chemokines between IPTi treatment groups for each cross-sectional visit.

			Unadjusted geometric mean					Unadjusted linear regression*		Adjusted linear regression*,^†^	
			**Placebo**		**SP**		***P *value^‡^**		***P *value**		***P *value**
**Cytokines and chemokines**		**Age (months)**	**GM**	**95% CI**	**GM**	**95% CI**		**Proportional difference^§^**		**Proportional difference**	

Intracellular (% producing lymphocytes)	IFN-γ	5	0.17	0.13 - 0.23	0.13	0.10 - 0.18	0.210	0.77	0.207	0.80	0.293
		
		9	0.05	0.04 - 0.07	0.05	0.04 - 0.07	0.994	1.00	0.994	1.01	0.965
		
		12	0.07	0.05 - 0.11	0.05	0.03 - 0.08	0.348	0.75	0.344	0.76	0.363
		
		24	0.05	0.04 - 0.08	0.04	0.03 - 0.06	0.395	0.80	0.391	0.82	0.433
	
	IL-4	5	0.96	0.81 - 1.12	0.94	0.80 - 1.10	0.864	0.98	0.863	1.00	0.995
		
		9	0.87	0.77 - 0.99	0.82	0.71 - 0.95	0.544	0.94	0.542	0.94	0.546
		
		12	0.80	0.64 - 0.99	0.91	0.71 - 1.17	0.427	1.14	0.422	1.13	0.453
		
		24	0.86	0.70 - 1.04	0.82	0.65 - 1.04	0.814	0.96	0.812	0.97	0.848
	
	IL-10	5	0.37	0.30 - 0.45	0.36	0.31 - 0.43	0.933	0.99	0.933	1.01	0.922
		
		9	0.15	0.12 - 0.19	0.18	0.15 - 0.21	0.285	1.16	0.282	1.15	0.294
		
		12	0.23	0.17 - 0.31	0.22	0.17 - 0.31	0.886	0.97	0.885	0.96	0.852
		
		24	0.32	0.25 - 0.42	0.35	0.27 - 0.45	0.667	1.08	0.664	1.07	0.695

Plasma (pg/ml)	IL-2	5	16.17	12.31 - 21.24	17.25	13.04 - 22.82	0.742	1.07	0.741	1.12	0.569
		
		9	12.99	9.48 - 17.79	12.17	9.35 - 15.83	0.752	0.94	0.750	0.93	0.742
		
		12	8.56	6.34 - 11.56	7.17	5.22 - 9.86	0.424	0.84	0.422	0.84	0.438
		
		24	7.09	5.08 - 9.90	5.36	3.83 - 7.49	0.241	0.76	0.237	0.76	0.254
	
	IL-12	5	2.36	1.80 - 3.10	1.87	1.34 - 2.61	0.280	0.79	0.277	0.79	0.284
		
		9	2.37	1.74 - 3.22	1.54	1.21 - 1.96	0.030	0.65	0.030	0.66	0.030
		
		12	1.43	1.14 - 1.78	1.31	1.08 - 1.58	0.550	0.92	0.548	0.93	0.596
		
		24	2.35	1.83 - 3.01	2.22	1.74 - 2.83	0.747	0.95	0.745	0.97	0.844
	
	IFN-γ	5	156.50	105.27-232.66	147.8	98.96 - 220.93	0.842	0.94	0.841	0.97	0.928
		
		9	145.93	93.78 - 227.08	115.0	75.84 - 174.44	0.437	0.79	0.435	0.79	0.434
		
		12	77.61	51.44 - 117.10	55.47	35.17 - 87.47	0.280	0.71	0.278	0.71	0.267
		
		24	128.29	85.71 - 192.03	79.89	51.45 - 124.06	0.116	0.62	0.113	0.62	0.106
	
	IL-1β	5	35.03	25.11 - 48.87	41.15	28.66 - 59.09	0.516	1.17	0.514	1.16	0.547
		
		9	11.61	8.50 - 15.86	11.45	8.46 - 15.50	0.950	0.99	0.950	0.99	0.957
		
		12	13.53	9.70 - 18.86	13.98	10.54 - 18.55	0.880	1.03	0.879	1.04	0.869
		
		24	20.05	13.99 - 28.73	17.64	12.12 - 25.69	0.625	0.88	0.622	0.86	0.566
	
	IL-6	5	99.22	72.21 - 136.34	127.6	97.52 - 166.95	0.231	1.29	0.228	1.27	0.254
		
		9	59.24	43.36 - 80.95	56.47	41.93 - 76.05	0.825	0.95	0.824	0.95	0.810
		
		12	76.28	54.51 - 106.75	70.96	51.65 - 97.48	0.756	0.93	0.755	0.93	0.772
		
		24	82.64	55.45 - 123.14	80.68	54.88 - 118.62	0.931	0.98	0.931	0.97	0.907
	
	TNF	5	53.64	38.35 - 75.02	54.50	40.40 - 73.51	0.944	1.02	0.943	1.00	0.991
		
		9	31.91	22.10 - 46.06	27.30	19.11 - 39.00	0.546	0.86	0.544	0.85	0.534
		
		12	21.72	15.16 - 31.12	20.49	14.29 - 29.37	0.820	0.94	0.819	0.95	0.832
		
		24	12.36	7.72 - 19.81	9.86	6.58 - 14.76	0.468	0.80	0.464	0.78	0.430
	
	IL-4	5	3.95	2.83 - 5.52	5.64	4.18 - 7.62	0.117	1.43	0.115	1.49	0.078
		
		9	2.96	1.99 - 4.39	3.64	2.69 - 4.93	0.403	1.23	0.401	1.23	0.406
		
		12	2.49	1.81 - 3.44	2.65	1.93 - 3.64	0.785	1.06	0.783	1.08	0.747
		
		24	1.41	0.98 - 2.04	1.44	1.09 - 1.90	0.939	1.02	0.938	1.01	0.972
	
	IL-5	5	2.65	2.14 - 3.28	2.66	2.16 - 3.29	0.966	1.01	0.966	1.03	0.833
		
		9	2.57	1.97 - 3.35	2.16	1.73 - 2.69	0.310	0.84	0.307	0.84	0.313
		
		12	2.05	1.68 - 2.50	1.62	1.32 - 2.00	0.109	0.79	0.106	0.80	0.116
		
		24	2.15	1.87 - 2.46	1.97	1.75 - 2.22	0.349	0.92	0.346	0.92	0.351
	
	IL-13	5	0.81	0.66 - 0.99	0.78	0.61 - 0.99	0.811	0.96	0.810	0.97	0.858
		
		9	0.71	0.54 - 0.92	0.57	0.46 - 0.71	0.212	0.81	0.209	0.81	0.213
		
		12	0.56	0.45 - 0.69	0.41	0.32 - 0.52	0.052	0.73	0.050	0.74	0.068
		
		24	1.36	1.11 - 1.67	1.36	1.11 - 1.67	0.993	1.00	0.993	1.01	0.930
	
	IL-10	5	3.77	3.01 - 4.72	4.20	3.32 - 5.32	0.510	1.11	0.508	1.14	0.431
		
		9	3.94	3.00 - 5.16	3.56	2.94 - 4.30	0.540	0.90	0.538	0.89	0.483
		
		12	2.74	2.21 - 3.40	2.45	1.99 - 3.01	0.451	0.89	0.449	0.91	0.536
		
		24	2.53	2.00 - 3.19	2.40	1.80 - 3.20	0.781	0.95	0.779	1.00	0.997
	
	IL-7	5	3.79	3.13 - 4.59	3.37	2.80 - 4.04	0.379	0.89	0.376	0.91	0.465
		
		9	3.98	3.13 - 5.05	3.20	2.71 - 3.79	0.144	0.81	0.142	0.81	0.144
		
		12	3.01	2.55 - 3.54	2.33	1.96 - 2.79	0.038	0.78	0.037	0.78	0.043
		
		24	2.41	2.11 - 2.75	2.29	2.02 - 2.59	0.560	0.95	0.556	0.94	0.519
	
	IL-17	5	4.73	3.47 - 6.45	4.77	3.70 - 6.16	0.968	1.01	0.968	0.99	0.950
		
		9	3.72	2.86 - 4.84	2.87	2.22 - 3.72	0.167	0.77	0.164	0.78	0.169
		
		12	3.01	2.32 - 3.91	2.93	2.29 - 3.76	0.887	0.97	0.886	0.98	0.890
		
		24	5.56	4.37 - 7.08	4.28	3.26 - 5.61	0.152	0.77	0.149	0.76	0.134
	
	G-CSF	5	17.84	14.28 - 22.29	16.05	12.87 - 20.01	0.502	0.90	0.499	0.91	0.540
		
		9	13.85	10.91 - 17.57	12.32	9.98 - 15.19	0.464	0.89	0.462	0.89	0.467
		
		12	10.48	8.17 - 13.44	11.11	9.18 - 13.44	0.712	1.06	0.710	1.07	0.647
		
		24	27.96	23.58 - 33.16	22.21	19.14 - 25.76	0.044	0.79	0.042	0.79	0.043
	
	GM-CSF	5	18.29	11.87 - 28.19	19.95	13.03 - 30.54	0.777	1.09	0.776	1.12	0.712
		
		9	18.34	11.82 - 28.46	12.78	8.34 - 19.59	0.243	0.70	0.240	0.70	0.243
		
		12	10.62	7.05 - 16.00	8.10	5.37 - 12.24	0.356	0.76	0.353	0.77	0.363
		
		24	10.91	7.05 - 16.87	8.77	6.23 - 12.35	0.433	0.80	0.429	0.81	0.437
	
	MCP-1	5	43.17	36.47 - 51.10	46.18	38.41 - 55.51	0.594	1.07	0.592	1.08	0.547
		
		9	41.14	33.21 - 50.95	41.49	35.24 - 48.85	0.949	1.01	0.949	1.01	0.969
		
		12	39.93	33.47 - 47.62	32.71	27.32 - 39.16	0.118	0.82	0.116	0.82	0.122
		
		24	56.52	48.17 - 66.32	50.18	41.82 - 60.22	0.330	0.89	0.326	0.89	0.332
	
	MIP-1β	5	565.32	474.10 - 674.10	693.5	603.63 - 796.78	0.070	1.23	0.069	1.18	0.131
		
		9	402.91	342.05 - 474.60	456.4	387.36 - 537.80	0.287	1.13	0.285	1.13	0.291
		
		12	487.27	413.65 - 573.99	583.6	482.50 - 705.98	0.157	1.20	0.155	1.20	0.153
		
		24	618.41	515.94 - 741.22	593.8	494.69 - 712.89	0.754	0.96	0.752	0.96	0.770
	
	IL-8	5	200.40	148.28 - 270.82	238.2	183.74 - 308.79	0.389	1.19	0.386	1.15	0.489
		
		9	126.87	99.27 - 162.15	139.2	108.84 - 178.11	0.597	1.10	0.596	1.10	0.592
		
		12	183.34	143.44 - 234.33	228.4	174.16 - 299.56	0.236	1.25	0.234	1.24	0.244
		
		24	208.38	150.71 - 288.13	170.5	123.01 - 236.25	0.386	0.82	0.382	0.81	0.345

The three analyses methods are presented and *P *values correspond to each one of these.

* Linear regression

^† ^Adjusted by previous episodes of clinical malaria

^‡ ^*T *test

^§ ^Proportional difference in cytokine/chemokine concentrations in SP-recipients in relation to placebo-recipients

Also, in general, no significant differences were found in plasma cytokine or chemokine responses between treatment groups. There were three isolated exceptions (IL-12 at 9 months, IL-7 at 12 months, G-CSF at 24 months) in which cases cytokine/chemokine concentrations were consistently higher in children who received placebo (Table 1), but associations were no longer statistically significant once adjusted for multiple comparisons. The complete raw data depicting levels of each cytokine and chemokine studied per age and treatment group is shown in Additional file 3, Figure S1.

### Factors associated with individual cytokine responses

Cytokine/chemokine levels varied according to the age of the child. All of them showed a decline from 5 to 9 months of age. Thereafter, some cytokines continued decreasing gradually throughout all measurements (plasma IL-2, TNF, IL-4, IL-10), others reached their highest levels at 24 months (plasma IL-12, IL-13, IL-17, MCP-1, G-CSF), and the remaining varied without any specific pattern (Table 2; Additional file 3, Figure S1).

**Table 2:** Intracellular and plasma cytokines and chemokines by age, analyzed by multivariate random-effect models estimated by stepwise procedure and adjusted by previous episodes, current infection, IPTi treatment, gender and neighborhood of residence.

Cytokines and chemokines		Age (months)	Proportional difference	95% CI	*P *value*	Overall *P *value
Intracellular	IFN-γ	5	1	-	-	< 0.0001
			
		9	0.36	0.26; 0.48	< 0.0001	
			
		12	0.41	0.29; 0.57	< 0.0001	
			
		24	0.32	0.23; 0.45	< 0.0001	
	
	IL-10	5	1	-	-	< 0.0001
			
		9	0.45	0.37; 0.55	< 0.0001	
			
		12	0.63	0.50; 0.78	< 0.0001	
			
		24	0.92	0.74; 1.15	0.4700	

Plasma	IL-2	5	1	-	-	< 0.0001
			
		9	0.74	0.58; 0.95	0.0193	
			
		12	0.47	0.37; 0.60	< 0.0001	
			
		24	0.38	0.29; 0.51	< 0.0001	
	
	IL-12	5	1	-	-	0.0001
			
		9	0.87	0.69; 1.10	0.2428	
			
		12	0.63	0.50; 0.79	0.0001	
			
		24	1.02	0.79; 1.31	0.9070	
	
	IFN-γ	5	1	-	-	< 0.0001
			
		9	0.84	0.59; 1.21	0.3510	
			
		12	0.43	0.30; 0.61	< 0.0001	
			
		24	0.69	0.47; 1.03	0.0703	
	
	IL-1β	5	1	-	-	< 0.0001
			
		9	0.30	0.22; 0.40	< 0.0001	
			
		12	0.36	0.27; 0.48	< 0.0001	
			
		24	0.51	0.38; 0.70	< 0.0001	
	
	IL-6	5	1	-	-	0.0001
			
		9	0.51	0.38; 0.68	< 0.0001	
			
		12	0.65	0.49; 0.87	0.0032	
			
		24	0.75	0.55; 1.03	0.0806	
	
	TNF	5	1	-	-	< 0.0001
			
		9	0.54	0.39; 0.73	0.0001	
			
		12	0.39	0.29; 0.53	< 0.0001	
			
		24	0.21	0.15; 0.30	< 0.0001	
	
	IL-4	5	1	-	-	< 0.0001
			
		9	0.69	0.53; 0.90	0.0073	
			
		12	0.54	0.41; 0.71	< 0.0001	
			
		24	0.31	0.23; 0.41	< 0.0001	
	
	IL-5	5	1	-	-	0.0001
			
		9	0.88	0.75; 1.04	0.1450	
			
		12	0.68	0.58; 0.81	< 0.0001	
			
		24	0.77	0.65; 0.93	0.0056	
	
	IL-13	5	1	-	-	< 0.0001
			
		9	0.77	0.64; 0.92	0.0045	
			
		12	0.58	0.49; 0.70	< 0.0001	
			
		24	1.59	1.30; 1.94	< 0.0001	
	
	IL-10	5	1	-	-	< 0.0001
			
		9	0.85	0.71; 1.03	0.0941	
			
		12	0.60	0.50; 0.73	< 0.0001	
			
		24	0.53	0.43; 0.66	< 0.0001	
	
	IL-7	5	1	-	-	< 0.0001
			
		9	0.99	0.86; 1.15	0.9244	
			
		12	0.74	0.64; 0.86	< 0.0001	
			
		24	0.66	0.56; 0.77	< 0.0001	
	
	IL-17	5	1	-	-	< 0.0001
			
		9	0.68	0.54; 0.85	0.0009	
			
		12	0.63	0.50; 0.79	0.0001	
			
		24	1.05	0.82; 1.35	0.6774	
	
	G-CSF	5	1	-	-	< 0.0001
			
		9	0.77	0.64; 0.91	0.0032	
			
		12	0.64	0.53; 0.76	< 0.0001	
			
		24	1.50	1.24; 1.81	< 0.0001	
	
	GM-CSF	5	1	-	-	< 0.0001
			
		9	0.78	0.56; 1.09	0.1525	
			
		12	0.49	0.35; 0.68	< 0.0001	
			
		24	0.52	0.36; 0.75	0.0004	
	
	MCP-1	5	1	-	-	< 0.0001
			
		9	0.92	0.80; 1.06	0.2724	
			
		12	0.80	0.70; 0.93	0.0025	
			
		24	1.22	1.04; 1.42	0.0156	
	
	MIP-1β	5	1	-	-	< 0.0001
			
		9	0.68	0.58; 0.80	< 0.0001	
			
		12	0.85	0.73; 1.00	0.0437	
			
		24	0.98	0.82; 1.16	0.7803	
	
	IL-8	5	1	-	-	0.0003
			
		9	0.60	0.47; 0.77	0.0001	
			
		12	0.94	0.73; 1.20	0.6171	
			
		24	0.89	0.68; 1.17	0.3991	

Intracellular IL-4 is not included (null model). The first *P *value corresponds to the comparison in between ages and the second corresponds to the overall comparison.

* Each age in relation to 5 months

In addition to age, plasma IL-10 levels were also associated with having had previous episodes of clinical malaria (proportional difference = 1.45 [1.14-1.86], *P *= 0.0028) and having current infection (proportional difference = 1.80 [1.41-2.29], *P *< 0.0001). IL-12 and IL-13 were also associated with having had previous malaria episodes (proportional difference = 1.35 [1.02-1.77], *P *= 0.0340; proportional difference = 1.32 [1.04-1.67], *P *= 0.0234). None of the other factors analyzed in the multivariate analyses (gender, neighborhood of residence, treatment) had any significant association with the cytokines assessed here.

### Correlations and ratios between cytokines

We observed no correlation between the magnitude of IL-4, IL-10 or IFN-γ responses measured intracellularly upon antigen-stimulation and plasma concentrations (data not shown). A few correlations were found between plasma cytokines when analyzed against each other (Additional file 4, Figure S2). Concentrations of Th1 (IL-2, IL-12, IFN-γ) and Th2 (IL-4, IL-5, IL-13) cytokines correlated moderately within themselves, and both these two types showed a moderate/high correlation in between them (Th1 vs Th2). Pro-inflammatory (IL-1β, IL-6, TNF, IL-17) and anti-inflammatory/regulatory (IL-10, IL-7) cytokines correlated moderately within themselves, and both these showed a moderate/low correlation between them (pro- vs anti-inflammatory). Chemokines G-CSF and GM-CSF both correlated moderately/highly to Th1 and Th2 type cytokines, whereas MCP-1 correlated with most anti-inflammatory cytokines and MIP-1 β and IL-8 correlated with most pro-inflammatory cytokines.

We examined whether there was a transition from Th2 to Th1 [55] or from anti-inflammatory to pro-inflammatory cytokines with age in early infancy by analyzing the ratios. Overall, Th1 responses were higher than Th2 responses over the 2 years of life (Additional file 3, Figure S1), and the ratio of IFN-γ:IL-4 increased from 5 to 24 months (Table 3). When data was stratified by treatment, IFN-γ:IL-4 ratios were higher for placebo than for SP recipients at all ages (Table 3), particularly at age 24 months coinciding with the highest prevalence of *P. falciparum *infection (7.7% at 5 months, 13.5% at 9 months, 8% at 12 months and 26.6% at 24 months). Overall, the pro-inflammatory responses were higher than anti-inflammatory responses over the 2 years of life (Additional file 3, Figure S1), and the ratio of TNF:IL-10 decreased from 5 to 24 months (Table 3).

**Table 3:** Ratios between prototype Th1:Th2 and pro-: anti-inflammatory cytokines.

		5 months		9 months		12 months		24 months	
		Ratio	95% CI	Ratio	95% CI	Ratio	95% CI	Ratio	95% CI
	Overall	32	31.73 - 32.27	39	38.73 - 39.27	25	24.69 - 25.31	71	70.75 - 71.25
	
**IFN-γ:IL-4**	SP	26	25.64 - 26.36	32	31.62 - 32.38	21	20.61 - 21.39	56	55.62 - 56.38
	
	Placebo	40	39.62 - 40.38	49	48.62 - 49.38	31	30.54 - 31.46	91	90.68 - 91.32

	Overall	14	13.80 - 14.20	8	7.79 - 8.21	8	7.78 - 8.22	4	3.69 - 4.31
	
**TNF:IL-10**	SP	13	12.71 - 13.29	8	7.73 - 8.27	8	7.70 - 8.30	4	3.58 - 4.42
	
	Placebo	14	13.73 - 14.27	8	7.69 - 8.31	8	7.67 - 8.33	5	4.55 - 5.45

### Individual cytokines and incidence of malaria

Elevated cytokine responses, both antigen-specific and non-specific, were not associated with a reduction in the subsequent incidence of clinical malaria in unadjusted or adjusted analyses (Table 4). In some cases there was an association between higher cytokine levels and higher malaria incidence and this was manifested at certain age intervals (5-12 months or 12-24 months). In particular, intracellular IFN-γ and IL-10 production by CD3+ cells was associated with higher incidence of malaria during the first year of age but not in the second year (Table 4). Adjusting for previous episodes did not affect these results. After Bonferroni correction for multiple comparisons, a non significant trend association was still observed (Table 4).

**Table 4:** Intracellular and plasma cytokines and chemokines and their association with incidence of malaria during the first and second years of age.

		5 - 12 months												12 - 24 months											
		Tertiles*						**Two-fold increment^†^**						**Tertiles**						**Two-fold increment**					

		**Unadjusted**			**Adjusted^‡^**			**Unadjusted**			**Adjusted**			**Unadjusted**			**Adjusted**			**Unadjusted**			**Adjusted**		

**Cytokines and chemokines**		**IRR**	**95% CI**	***P *value^§^**	**IRR**	**95% CI**	***P *value**	**IRR**	**95% CI**	***P *value**	**IRR**	**95% CI**	***P *value**	**IRR**	**95% CI**	***P *value**	**IRR**	**95% CI**	***P *value**	**IRR**	**95% CI**	***P *value**	**IRR**	**95% CI**	***P *value**

	IFN-γ	3.24	1.13 - 9.33	0.052	4.62	1.58 - 13.47	**0.004**	1.21	1.03 - 1.43	0.021	1.26	1.07 - 1.49	**0.003**	0.95	0.38 - 2.39	0.635	0.68	0.32 - 1.45	0.533	0.97	0.84 - 1.11	0.639	0.96	0.85 - 1.08	0.5226
	
Intracellular	IL-4	1.49	0.64 - 3.48	0.645	1.31	0.62 - 2.77	0.783	1.19	0.90 - 1.58	0.212	1.17	0.91 - 1.49	0.213	0.53	0.20 - 1.37	0.404	0.78	0.35 - 1.72	0.816	0.81	0.60 - 1.10	0.178	0.95	0.75 - 1.20	0.6635
	
	IL-10	3.11	1.12 - 8.67	0.086	3.40	1.30 - 8.89	0.033	1.32	1.04 - 1.67	0.022	1.38	1.11 - 1.71	**0.004**	0.97	0.38 - 2.47	0.989	1.39	0.68 - 2.82	0.239	0.98	0.80 - 1.20	0.833	0.96	0.82 - 1.12	0.5904
	
	IL-2	2.71	0.94 - 7.83	0.131	2.13	0.83 - 5.42	0.219	1.19	0.97 - 1.45	0.083	1.11	0.95 - 1.29	0.200	1.54	0.66 - 3.59	0.423	1.98	1.01 - 3.90	0.055	1.12	0.98 - 1.29	0.090	1.11	1.00 - 1.24	0.051
	
	IL-12	1.38	0.56 - 3.41	0.309	1.53	0.71 - 3.30	0.080	1.10	0.91 - 1.34	0.330	1.06	0.90 - 1.25	0.453	1.63	0.63 - 4.21	0.528	1.87	0.91 - 3.82	0.223	1.09	0.88 - 1.35	0.422	1.10	0.92 - 1.32	0.266
	
	IFN-γ	0.92	0.37 - 2.32	0.929	0.99	0.44 - 2.21	0.378	1.00	0.89 - 1.13	0.992	0.98	0.89 - 1.08	0.706	1.28	0.56 - 2.93	0.836	3.60	1.79 - 7.23	**0.001**	1.05	0.95 - 1.16	0.316	1.16	1.07 - 1.26	**0.0004**
	
	IL-1β	1.42	0.43 - 4.73	0.654	1.24	0.41 - 3.71	0.653	1.00	0.84 - 1.19	0.975	1.02	0.87 - 1.20	0.813	1.52	0.63 - 3.68	0.050	2.11	1.11 - 4.01	0.009	1.11	0.94 - 1.31	0.194	1.18	1.04 - 1.33	0.011
	
	IL-6	1.03	0.40 - 2.69	0.967	1.05	0.45 - 2.45	0.967	1.00	0.85 - 1.18	0.979	1.02	0.88 - 1.19	0.747	1.52	0.67 - 3.46	0.609	1.86	0.95 - 3.63	0.184	1.10	0.95 - 1.26	0.194	1.16	1.03 - 1.30	0.013

Plasma	TNF	1.53	0.52 - 4.48	0.716	2.08	0.80 - 5.43	0.116	1.13	0.95 - 1.34	0.166	1.12	0.97 - 1.30	0.117	1.00	0.43 - 2.33	0.926	1.37	0.68 - 2.77	0.580	1.05	0.94 - 1.19	0.390	1.06	0.96 - 1.16	0.243
	
	IL-4	1.28	0.48 - 3.41	0.506	1.21	0.53 - 2.78	0.516	1.08	0.94 - 1.24	0.253	1.07	0.95 - 1.20	0.271	1.83	0.82 - 4.07	0.168	2.26	1.19 - 4.27	0.041	1.11	0.98 - 1.25	0.093	1.16	1.05 - 1.29	**0.004**
	
	IL-5	1.67	0.58 - 4.80	0.618	1.74	0.69 - 4.37	0.485	1.13	0.87 - 1.48	0.347	1.05	0.84 - 1.32	0.652	1.90	0.77 - 4.69	0.329	2.80	1.43 - 5.49	**0.001**	1.15	0.89 - 1.49	0.289	1.39	1.13 - 1.70	**0.002**
	
	IL-13	1.35	0.46 - 3.92	0.527	1.70	0.66 - 4.37	0.114	1.22	0.92 - 1.62	0.157	1.19	0.94 - 1.50	0.132	3.33	1.21 - 9.15	0.038	3.26	1.45 - 7.32	0.016	1.42	1.11 - 1.81	**0.005**	1.32	1.07 - 1.62	0.006
	
	IL-10	3.36	1.27 - 8.87	0.035	3.06	1.21 - 7.76	0.025	1.28	1.04 - 1.57	0.016	1.17	0.99 - 1.38	0.059	3.61	1.60 - 8.13	0.009	2.30	1.19 - 4.44	0.045	1.43	1.16 - 1.75	**0.0004**	1.25	1.06 - 1.47	0.006
	
	IL-7	1.77	0.62 - 5.04	0.529	1.41	0.56 - 3.52	0.594	1.26	0.92 - 1.73	0.137	1.10	0.85 - 1.43	0.456	1.56	0.62 - 3.96	0.242	2.16	1.09 - 4.30	0.029	1.20	0.88 - 1.65	0.245	1.34	1.04 - 1.72	0.019
	
	IL-17	0.67	0.25 - 1.83	0.622	0.76	0.33 - 1.74	0.551	1.01	0.83 - 1.23	0.914	1.04	0.88 - 1.22	0.681	0.78	0.30 - 2.00	0.055	0.80	0.36 - 1.77	0.278	0.97	0.79 - 1.20	0.785	0.92	0.78 - 1.09	0.342
	
	G-CSF	1.83	0.64 - 5.27	0.439	2.42	0.95 - 6.20	0.074	1.25	0.95 - 1.64	0.102	1.21	0.96 - 1.53	0.085	2.41	0.90 - 6.46	0.207	3.10	1.46 - 6.56	0.013	1.25	1.00 - 1.55	0.048	1.35	1.13 - 1.63	**0.001**
	
	GM-CSF	1.19	0.41 - 3.42	0.945	1.19	0.48 - 2.97	0.490	1.05	0.92 - 1.21	0.475	1.04	0.93 - 1.16	0.517	1.63	0.64 - 4.10	0.187	2.41	1.16 - 5.05	0.011	1.11	0.99 - 1.26	0.077	1.13	1.03 - 1.24	0.010
	
	MCP-1	3.01	1.18 - 7.63	0.064	3.16	1.37 - 7.25	0.015	1.64	1.21 - 2.23	**0.001**	1.66	1.29 - 2.15	**< 0.001**	3.07	1.34 - 7.06	0.008	2.85	1.43 - 5.69	0.007	1.31	1.02 - 1.69	0.038	1.36	1.11 - 1.67	**0.003**
	
	MIP-1β	0.52	0.20 - 1.35	0.113	0.83	0.34 - 2.07	0.341	0.92	0.67 - 1.27	0.620	1.10	0.80 - 1.51	0.571	1.50	0.67 - 3.40	0.396	0.98	0.50 - 1.94	0.329	0.98	0.78 - 1.24	0.859	0.93	0.76 - 1.13	0.430
	
	IL-8	0.68	0.27 - 1.71	0.646	0.77	0.34 - 1.73	0.809	0.95	0.79 - 1.14	0.580	0.98	0.82 - 1.16	0.781	0.72	0.32 - 1.62	0.402	0.61	0.30 - 1.22	0.100	0.95	0.80 - 1.12	0.543	0.97	0.84 - 1.11	0.633

For each time interval (5 to 12 months and 12 to 24 months) *P *values correspond to each one of the analyses methods used.

* Incidence rate ratio in high tertile relative to low tertile

^† ^Incidence rate ratio in two-fold increment

^‡ ^Adjusted by treatment group, gender, previous episodes of clinical malaria, current infection, neighborhood

^§ ^Negative binomial regression model using likelihood ratio test. In bold, *P *values that remained statistically significant (P < 0.05) or border line significant (P < 0.095) after Bonferroni correction for multiple comparisons. Corrected *P *values (in order from top to bottom, left to right): intracellular IFN-γ, 0.083, 0.052; intracellular IL-10, 0.076; plasma IFN-γ, 0.022, 0.008; plasma IL4, 0.077; IL-5, 0.027, 0.031; IL-13, 0.093; plasma IL-10, 0.008; G-CSF, 0.018; MCP-1, 0.019, 0.002, 0.053.

Associations between individual plasma cytokines and incidence of malaria were observed throughout the two years, but mostly in the second year. High concentrations of IL-10 and MCP-1 in plasma were associated with higher malaria incidence in both time intervals and by all analysis methods (Table 4). High IL-13 was also positively associated with incidence of malaria as analysed by all methods but only during the second year. A few disperse significant associations for plasma IFN-γ, IL-1β, IL-4, IL-5, IL-7, G-CSF and GM-CSF were also observed during the second year, when adjusted the analyses by the variables included in the multivariate model; all were in the same direction of higher concentrations correlating with increased malaria risk (Table 4). After correction for multiple comparisons, some statistically significant associations remained for MCP-1, IFN-γ, IL-10 and IL-5 (Table 4).

### Multiple cytokines and incidence of malaria

We did a multiple regression analysis in which we used a stepwise procedure to determine the variables that were independently associated with incidence of malaria. All cytokines were included in the list of variables taken into account for the stepwise procedure. Significant associations were only observed between higher levels of intracellular IFN-γ at 5 months and higher malaria incidence in the follow up interval 5-12 months (IRR 1.28, 95% CI 1.03-1.58, *P *= 0.0234). Similarly, higher concentrations of plasma IFN-γ (IRR 1.15, 95% CI 1.05-1.27, *P *= 0.0040) and MCP-1 (IRR 1.60, 95% CI 1.22-2.10, *P *= 0.0007) at 12 months were associated with higher malaria risk during the 12-24 month period. In contrast, in this analysis higher plasma concentrations of IL-17 at 12 months were associated with lower malaria incidence during the interval 12-24 months (IRR 0.80, 95% CI 0.69-0.93, *P *= 0.0035). Other co-variates that were significantly associated with malaria incidence, independently of the cytokine concentrations, were having had a previous episode in the second year of life (IRR 4.63, 95% CI 2.59-8.26, *P *< 0.0001), and the child's neighborhood of residence in both time intervals (*P *< 0.0001).

When antibody variables from prior studies were also included in the stepwise model together with all cytokines and co-variates, significant associations were only observed for higher intracellular IFN-γ at 5 months and higher malaria incidence during the follow up interval 5-12 months (IRR 1.26, 95% CI 1.01-1.57, *P *= 0.0381). At 12 months, higher plasma IFN-γ (IRR 1.26, 95% CI 1.11-1.44, *P *= 0.0004) and IL-5 (IRR 1.27, 95% CI 1.04-1.55, *P *= 0.0203) correlated with higher incidence of malaria up to 24 months, while higher IL-17 plasma concentrations were associated with lower malaria risk during the 12-24 month period (IRR 0.84, 95% CI 0.71-0.98, *P *= 0.0300). Likewise, the additional variables that independently showed significant associations with malaria risk were having had a previous episode in the second year of life (IRR 3.16, 95% CI 1.64-6.09, *P *< 0.0006), and the neighborhood in both time intervals (*P *< 0.0001).

Finally, no significant differences in cytokine or chemokine levels were found in relation to parasite density (data not shown). Levels of cytokines or chemokines could not be analysed in relation to incidence of severe malaria due to its low incidence (Additional file 2, Table S2).

## Discussion

In the context of a clinical trial evaluating the safety and efficacy of IPTi-SP as a malaria control tool during infancy, we studied prospectively the cytokine responses to *P. falciparum *antigens and the concentrations of plasma cytokines and chemokines at multiple time points during a 2 year follow up period. The longitudinal design and the established demographic and morbidity surveillance systems allowed for a rigorous and complete documentation of clinical malaria cases that could provide insights into the acquisition of cytokine/chemokine responses in children living in a malaria endemic area, how they are influenced by age and *P. falciparum *exposure, and the correlation between these responses and incidence of symptomatic malaria.

One of the main findings of this study was that overall there were no differences in the magnitude of intracellular or plasma cytokines/chemokines between IPTi treatment groups, except for some plasma cytokines and chemokines in which children receiving placebo had higher concentrations than those receiving SP (IL-12 at 9 months, IL-7 at 12 months and G-CSF at 24 months). In these cases, we hypotesize that placebo recipients, who suffered more *P. falciparum *infections than SP recipients owing to the efficacy of IPTi [57] had more systemic immune activation and higher levels of some plasma cytokines. However, of these, only IL-12 was directly associated to previous episodes of malaria in the multivariate analysis; to our knowledge, there is no data in the literature on the influence of *P. falciparum *infection or malaria interventions in the concentrations of IL-7 or G-CSF in peripheral blood. The levels of these cytokines/chemokines at those time points were not clearly associated with subsequent incidence of malaria.

Among the factors that significantly affected the levels of cytokines (both intracellular and plasma), age was the most prominent. Without exception, all cytokines varied significantly in magnitude from the first visit to at least one of the following visits. In all cases, there was a decrease of cytokine values from 5 to 9 months, and some had the highest levels at 24 months. This fluctuation is not surprising as cross-sectional samplings were not scheduled to pick up bursts of production upon infection and most plasma cytokines' half-lives are short [34], thus may not represent a long lasting response. In addition, cytokine production is stimulated and regulated by many different factors within the cellular responses network, and while some are specific to infection, other are innate or are produced in response to acute or systemic inflammatory conditions.

In contrast to antibody responses that are strongly affected by past or present malaria infection [59], parasite exposure appeared to have a minor effect on the concentrations of cytokines. Plasma IL-10 increased significantly in children with previous episodes or current infection, and higher levels of IL-12 and IL-13 were also associated with previous episodes. Each of these cytokines belongs to a different family (immuno-regulatory, Th1 and Th2 respectively) therefore it seems that the limited effect of exposure to *P. falciparum *infection was generalized and not biased towards a specific type. Similar observations have been reported [9] where malaria patients responded to *P. falciparum *infected erythrocytes with significant increases in the percentage of IL-2, IFN-γ, and TNF, but also IL-10, positive cells.

We analyzed the correlations and ratios among some representative cytokine families, as they have synergistic and antagonistic effects on each other to regulate the immune system. First, we observed no correlation between intracellular and plasma IL-4, IL-10 or IFN-γ, which is not uncommon as they represent two different measurements. Intracellular cytokines were produced by CD3+ cells after antigen stimulation in vitro (recall response) whereas plasma cytokines reflect what is present ex-vivo in peripheral blood (non malaria specific), and may be produced by many types of cells. In another study [66], the strength of associations between serum and cellular cytokines varied greatly, suggesting that serum cytokines at best only weakly reflect peripheral blood cell cytokine production and balances. We observed a high ratio between prototype Th1 (IFN-γ) and Th2 (IL-4) cytokines at all ages, and this proportion increased when looking only at the placebo group possibly indicating more parasite exposure. We also observed the relative transition from Th2 to Th1 responses with age [69].

We also assessed whether cytokines and chemokines were associated with subsequent incidence of malaria, and what factors affected these associations. Overall, high cytokine responses were not associated with a reduced incidence of clinical malaria when analyzed individually. Only when all cytokines where analyzed together in relation to risk of malaria, a significant association towards a decreased risk was found for IL-17 in the second year of life. IL-17 is a Th17 cytokine and its relation to various infectious agents has been described [70]. However to our knowledge, there has not been any study reporting a role for IL- 17 in protection against human malaria.

In fact, the most common finding was that there were no associations, or that higher levels of some cytokines correlated with increased malaria incidence. In particular, IL-10 (intracellular and plasma) and MCP-1 were more consistently associated with incidence of malaria, and this association was not explained by age or previous episodes. IL-10, an immunoregulatory cytokine, is extensively reported in relation to malaria immunopathogenesis [9,12,20,26,27] and not so much associated with immunity [38]. Higher IL-10 has been associated with less effective parasite clearance [71]. To our knowledge nothing has been reported for chemokine MCP-1 in children in relation to immunity, and it does not correlate with any of the other cytokines associated with increased incidence of malaria. In pregnant women, MCP-1 concentrations were higher in the placentas of primiparous women (more susceptible to malaria) than in those of multiparous women [46].

In addition, IFN-γ (intracellular and plasma) and IL-13 were also associated with malaria incidence, but these were dependent on age and/or previous episodes. A few other disperse associations were observed during the second year for plasma IL-1β, IL-4, IL-5, IL-7, G-CSF, GM-CSF, when adjusted by previous episodes. These results suggest that the responses measured were not necessarily part of an acquired protective response, but rather could be interpreted as biomarkers of physiopathological processes. In other studies, IL-13, IL-4 and IL-5, Th2-type cytokines, have been associated with reduced parasite killing [24]. Production of IL- 4 but not IFN-γ by activated human T cells has been associated with elevated antibodies to malaria antigens [72], and consistent with this, our previous studies also found correlations between higher antibody levels and increased malaria risk (Dobaño et al. *in press*).

Responses associated with reduced malaria incidence would more likely be expected in the antigen-specific cytokines, as measured by ICS. Blood cells from donors in malaria endemic areas stimulated with *Plasmodium *antigens are known to activate many types of cells, with production of both IFN-γ (Th1 type) and IL-4 (Th2 type) [72]. IFN-γ is thought to be a central mediator of protective immune responses against blood stages of malaria [73]. Surprisingly, in our study, increased frequency of IFN-γ positive CD3+ cells at 5 months was associated with higher incidence of malaria up to 1 year. This is in contrast with other studies since IFN-γ is more often reported to confer protection [30,32-36] than pathology [9,12,74]. In most of these studies IFN-γ was measured in older children or adults and it is likely that the immature immune system of the infant responds differently. In the second year, however, there were no significant associations between intracellular cytokines and incidence of malaria. At a population level, memory-like IFN-γ responses have been measured following malaria infection [75] and it has been reported that IFN-γ responses are both more prevalent and of greater magnitude at the end of the rainy malaria transmission season [45], thus showing that IFN-γ might also be a marker of exposure.

The apparent predisposition of children with higher cytokine responses to increased malaria risk might also be influenced by genetic factors. Many studies have shown associations between genetic polymorphisms, immunoregulation, phenotypes and disease risk [76-91], but unfortunately this was not assessed in this study. Other factors not evaluated that could help explain these associations would be malnutrition [92,93], co-infection with HIV or other pathogens [94], or prenatal exposure [95]. The duration and/or the nature of antigen exposure *in utero *appears to govern the outcome with respect to neonatal immune responses, such that placental malaria induce antigen-specific IL-10-producing regulatory T cells that can inhibit Th1-type responses, while antigen-specific IFN-γ production predominate in babies born to mothers successfully treated for malaria during gestation [95]. Prenatal infection could thus contribute to the *P. falciparum*-specific IFN-γ and IL-10 response pattern in 5-month old children seen in this study.

## Conclusions

Our work has further confirmed that IPTi-SP is a safe intervention that does not compromise the development of a wide range of cytokines and chemokines thought to be major contributors to anti-malarial immunity in infancy. This study has also given some insights as to how these responses are acquired upon age and exposure to the *P. falciparum *parasite. Due to the multiple comparisons performed, isolated findings are interpreted with caution, and only those associations that showed consistency and biological plausibility are considered here as potential true findings. Despite the low and heterogeneous antigen-specific cytokine responses observed, infant field studies such as the current one help to advance the understanding of the relationship between innate and adaptive cellular immune responses. Eventually, an effective orchestration of both types of immune responses is necessary for the generation of an efficient and non-pathogenic resolution of the malarial disease.

## Abbreviations

APC: (allophycocyanin); CD: (cluster of differentiation); CI: (confidence interval); CV: (coefficient of variation); DTP/OPV/Hep B: (diphteria+tetanus+pertussis/oral polio vaccine/hepatitis B); EDTA: (ethylenediaminetetraacetic acid); EPI: (expanded program on immunization); FITC: (fluorescein isothiocyanate); FSC: (forward scatter); G-CSF: (granulocyte colony stimulating factor); GM-CSF: (granulocyte macrophage colony stimulating factor); GM: (geometric means); HIV: (human immunodeficiency virus); ICS: (intracellular cytokine staining); Ig: (immunoglobulin); IL: (interleukin); IFN: (interferon); IPTi: (Intermittent Preventive Treatment in infants); IRR: (incidence rate ratio); MCP: (monocyte chemotactic protein); MCAF: (monocyte chemotactic and activating factor); MIP: (macrophage inflammatory protein); *P. falciparum*: (*Plasmodium falciparum*); PBS: (phosphate buffered saline); PCR: (polymerase chain reaction); PE: (phycoerythrin); PerCP: (Peridinin chlorophyll protein); PYAR: (person years at risk); SSC: (side scatter); SP: (sulfadoxine-pyrimethamine); TGF: (transforming growth factor); Th: (T helper); TNF: (tumor necrosis factor); WBA: (whole blood assay).

## Authors' contributions

PLA, CM, EM and PA carried out the IPTi clinical trial. PLA, CM and CD conceived, designed and coordinated the study. DQ, TN, ES and CD participated in the sample collection and processing. CD and DLD designed and supervised the immunoassays. DQ and LP carried out the immunoassays. LQ, SS and JJA performed the statistical analyses. DQ and CD drafted the first version of the manuscript. All authors read and approved the final manuscript.

## Supplementary Material

Additional file 1 **Table S1. Definition of the distribution of tertiles for cytokine production: low tertile (minimum to tertile cut-off 1), medium tertile (tertile cut-off 1 to tertile cut-off 2), and high tertile (tertile cut-off 2 to maximum)**. Click here for file

Additional file 2 **Table S2**. **Description of the clinical and parasitological characteristics of the groups of children receiving IPTi with SP or placebo in whom cytokine and chemokine responses were evaluated**. This description includes episodes of clinical malaria between 5 and 12 months of age, and between 12 and 24 months of age, which constituted the two follow up periods defined in the immunology study ancillary within the IPTi clinical trial conducted in Manhiça. Click here for file

Additional file 3 **Figure S1. Intracellular and plasma cytokines and chemokines in Mozambican infants receiving IPTi with SP (right) or placebo (left), at 5, 9 12 and 24 months. Cytokine values (Y axis) are expressed as % producing lymphocytes for intracellular cytokines, and as pg/ml for plasma cytokines/chemokines**. In the weighted scatter plots the area of the symbol is proportional to the number of observations. Geometric mean and 95% confidence intervals are indicated by horizontal continuous and discontinuous lines respectively. Click here for file

Additional file 4 **Figure S2. A) Correlation among cytokines, B) Correlation coefficients and strength of correlation**. Click here for file
