# Supplementary material for: Intermittent preventive treatment with sulfadoxine-pyrimethamine does not modify plasma cytokines and chemokines or intracellular cytokine responses to Plasmodium falciparum in Mozambican Children
Source: BMC Immunol. 2012 Jan 26;13:5. doi: 10.1186/1471-2172-13-5 (PMC3398260; doi:10.1186/1471-2172-13-5)
Supplement: Additional file 1 — Table S1. Definition of the distribution of tertiles for cytokine production: low tertile (minimum to tertile cut-off 1), medium tertile (tertile cut-off 1 to tertile cut-off 2), and high tertile (tertile cut-off 2 to maximum). [file 1471-2172-13-5-S1.DOC]

# Additional file 1, Table S1

Definition of the distribution of tertiles for cytokine production: low tertile (minimum to tertile cut-off 1), medium tertile (tertile cut-off 1 to tertile cut-off 2), and high tertile (tertile cut-off 2 to maximum).

| **Cytokines and chemokines** | | **Tertile cut-off 1** | **Tertile cut-off 2** |
| --- | --- | --- | --- |
| Intracellular | IFN-γ | 0.05 | 0.17 |
|  | IL-4 | 0.66 | 1.235 |
|  | IL-10 | 0.19 | 0.42 |
| Plasma | IL-2 | 6.35 | 21.24 |
|  | IL-12 | 1 | 2.71 |
|  | IFN-γ | 78.18 | 315.95 |
|  | IL-1β | 8.44 | 29.07 |
|  | IL-6 | 46.42 | 160.98 |
|  | TNF | 15.73 | 65.94 |
|  | IL-4 | 1.05 | 5.98 |
|  | IL-5 | 1.52 | 2.83 |
|  | IL-13 | 0.53 | 1.17 |
|  | IL-10 | 1.76 | 4.36 |
|  | IL-7 | 2.055 | 4.01 |
|  | IL-17 | 1.12 | 8.23 |
|  | G-CSF | 10.62 | 22.3 |
|  | GM-CSF | 4.83 | 38.12 |
|  | MCP-1 | 31.93 | 65.52 |
|  | MIP-1β | 357.22 | 902.45 |
|  | IL-8 | 101.46 | 366.74 |
