# Supplementary material for: Intermittent preventive treatment with sulfadoxine-pyrimethamine does not modify plasma cytokines and chemokines or intracellular cytokine responses to Plasmodium falciparum in Mozambican Children
Source: BMC Immunol. 2012 Jan 26;13:5. doi: 10.1186/1471-2172-13-5 (PMC3398260; doi:10.1186/1471-2172-13-5)
Supplement: Additional file 2 — Table S2. Description of the clinical and parasitological characteristics of the groups of children receiving IPTi with SP or placebo in whom cytokine and chemokine responses were evaluated. This description includes episodes of clinical malaria between 5 and 12 months of age, and between 12 and 24 months of age, which constituted the two follow up periods defined in the immunology study ancillary within the IPTi clinical trial conducted in Manhiça. [file 1471-2172-13-5-S2.DOC]

# Additional file 2, Table S2

Description of the clinical and parasitological characteristics of the groups of children receiving IPTi with SP or placebo in whom cytokine and chemokine responses were evaluated. This description includes episodes of clinical malaria between 5 and 12 months of age, and between 12 and 24 months of age, which constituted the two follow up periods defined in the immunology study ancillary within the IPTi clinical trial conducted in Manhiça.

|  | **SP** | **Placebo** |
| --- | --- | --- |
|  |  |  |
| Incidence of malaria (episodes per PYAR) |  |  |
| 5 to 12 months of age | 0.49 | 0.50 |
| 12 to 24 months of age | 0.64 | 0.77 |
|  |  |  |
| Number of episodes per child (5 to 12 months of age) |  |  |
| 0 episodes | 130 | 114 |
| 1 episode | 13 | 16 |
| ≥2 episodes | 12 | 10 |
|  |  |  |
| Number of episodes per child (12 to 24 months of age) |  |  |
| 0 episodes | 99 | 86 |
| 1 episode | 14 | 14 |
| ≥2 episodes | 20 | 22 |
|  |  |  |
| *Plasmodium falciparum* density (parasites/μl): mean |  |  |
| (SD, min, max) |  |  |
| 5 to 12 months of age | 17156 | 10018 |
|  | (26002, 152 150353) | (22167, 26, 238160) |
| 12 to 24 months of age | 20725 | 33999 |
|  | (37418, 76, 288889) | (52600, 219, 391268) |
|  |  |  |
| Severe malaria episodes (number) |  |  |
| 5 to 12 months of age | 2 | 1 |
| 12 to 24 months of age | 1 | 3 |

PYAR = person years at risk

SD = standard deviation; min = minimum; max = maximum
