# Supplementary figures and images for: Intermittent preventive treatment with sulfadoxine-pyrimethamine does not modify plasma cytokines and chemokines or intracellular cytokine responses to Plasmodium falciparum in Mozambican Children
Source: BMC Immunol. 2012 Jan 26;13:5. doi: 10.1186/1471-2172-13-5 (PMC3398260; doi:10.1186/1471-2172-13-5)

**Additional file 3, Figure S1.**


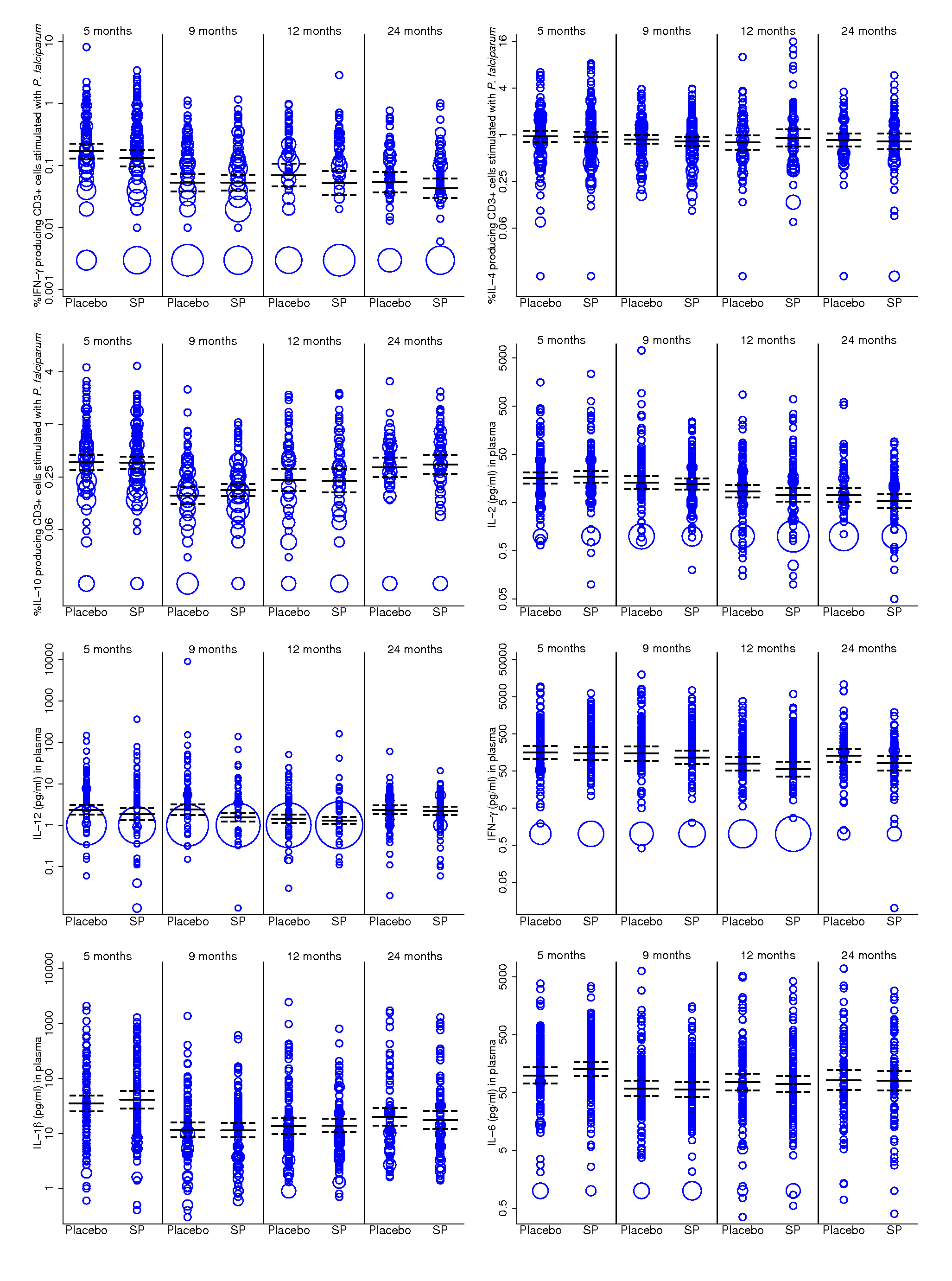


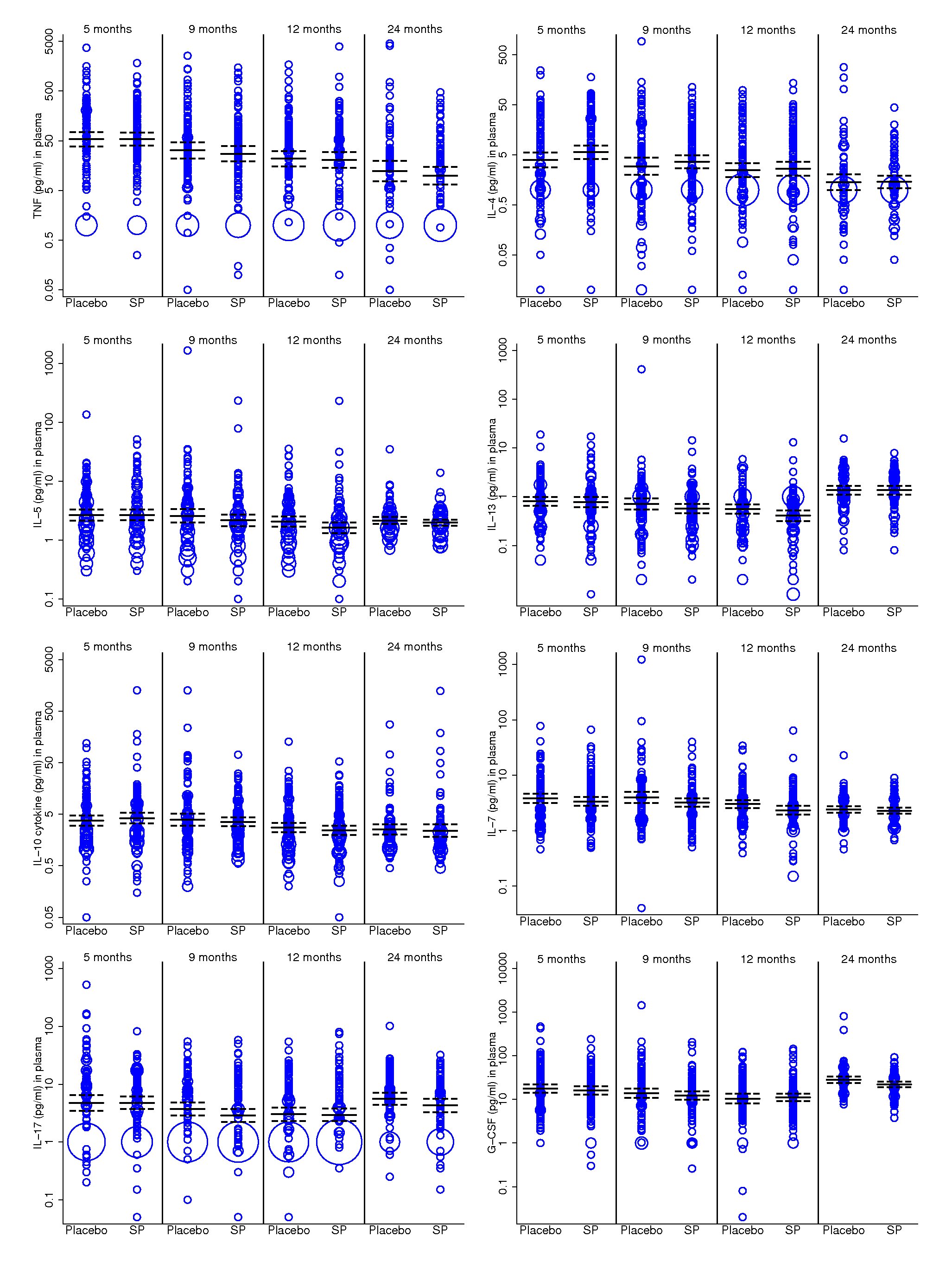


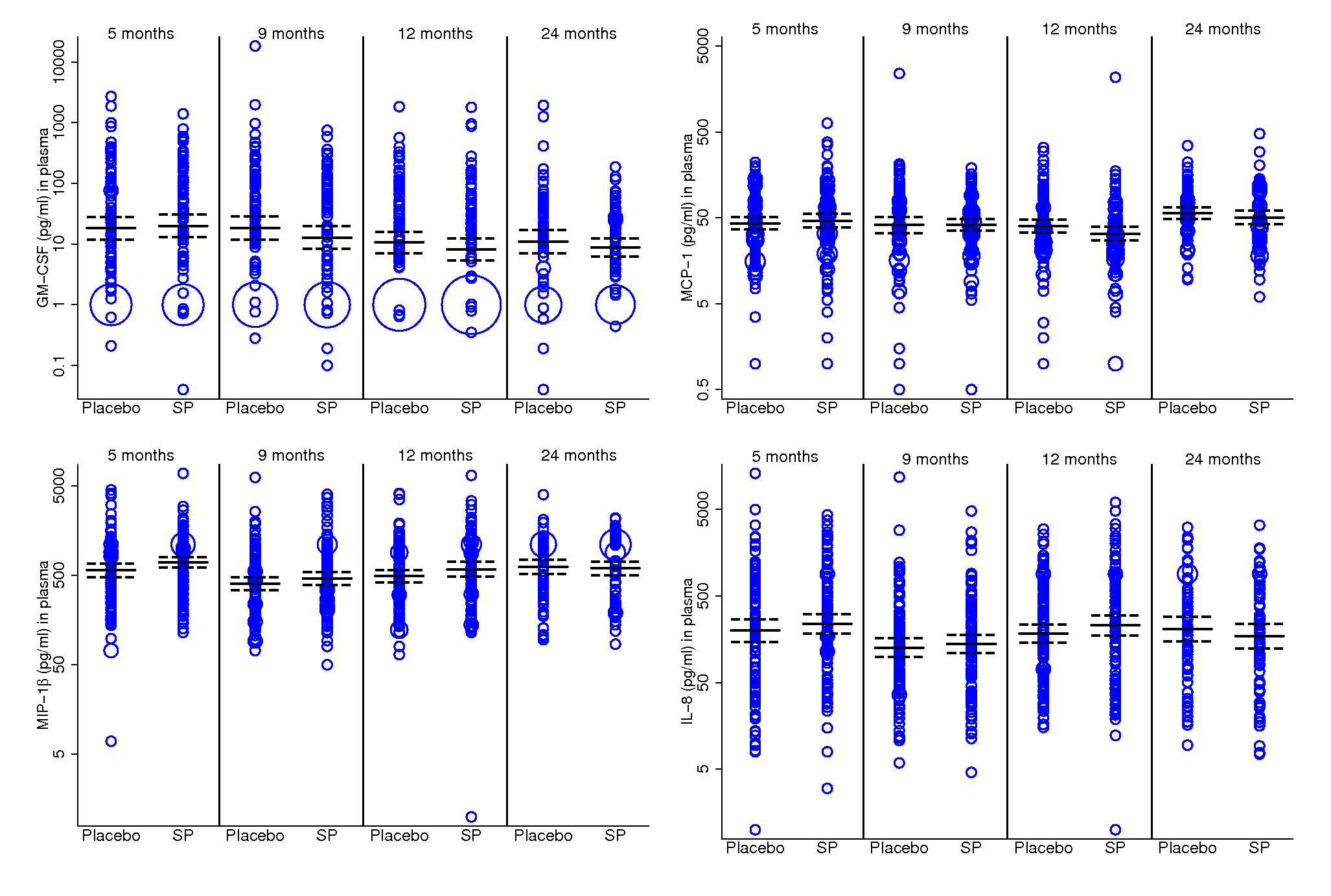

Supplement: Additional file 3 — Figure S1. Intracellular and plasma cytokines and chemokines in Mozambican infants receiving IPTi with SP (right) or placebo (left), at 5, 9 12 and 24 months. Cytokine values (Y axis) are expressed as % producing lymphocytes for intracellular cytokines, and as pg/ml for plasma cytokines/chemokines. In the weighted scatter plots the area of the symbol is proportional to the number of observations. Geometric mean and 95% confidence intervals are indicated by horizontal continuous and discontinuous lines respectively. [file 1471-2172-13-5-S3.DOC]
