# Supplementary material for: Intermittent preventive treatment with sulfadoxine-pyrimethamine does not modify plasma cytokines and chemokines or intracellular cytokine responses to Plasmodium falciparum in Mozambican Children
Source: BMC Immunol. 2012 Jan 26;13:5. doi: 10.1186/1471-2172-13-5 (PMC3398260; doi:10.1186/1471-2172-13-5)
Supplement: Additional file 4 — Figure S2. A) Correlation among cytokines, B) Correlation coefficients and strength of correlation. [file 1471-2172-13-5-S4.DOC]

**Additional file 4, Figure S2.** A: Correlation among cytokines; B: Correlation coefficients and strength of correlation.

**A)**

**
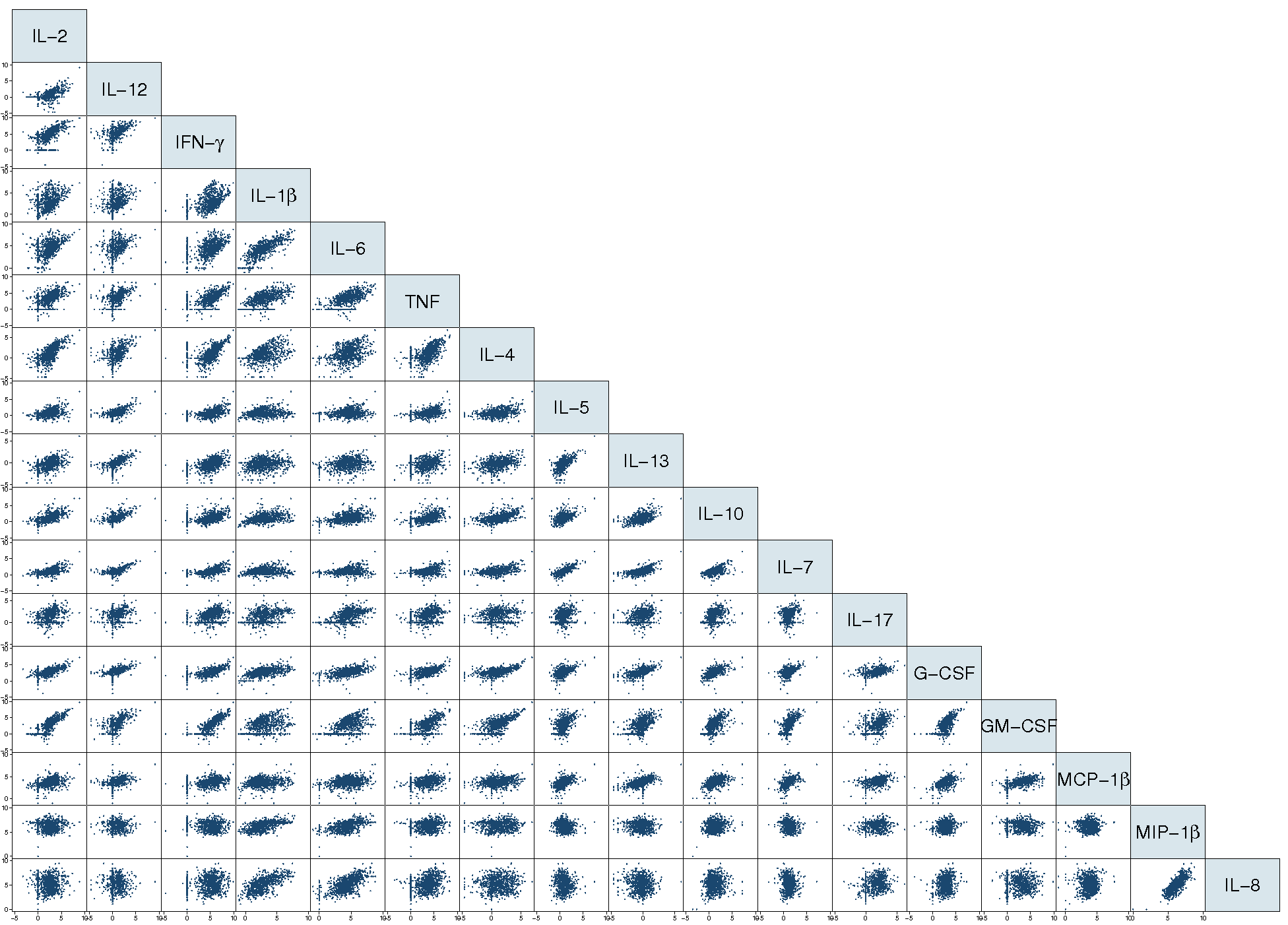
**

**B)**
